# Inflammation and immunity in liver homeostasis and disease: a nexus of hepatocytes, nonparenchymal cells and immune cells

**DOI:** 10.1038/s41423-025-01313-7

**Published:** 2025-07-01

**Authors:** Enis Kostallari, Robert F. Schwabe, Adrien Guillot

**Affiliations:** 1 Division of Gastroenterology and Hepatology, Mayo Clinic, Rochester, MN USA; 2 Department of Biochemistry and Molecular Biology, Mayo Clinic, Rochester, MN USA; 3 Department of Medicine, Columbia University, New York, NY USA; 4 Columbia University Digestive and Liver Disease Research Center, New York, NY USA; 5 Institute of Human Nutrition, New York, NY USA; 6 Charité—Universitätsmedizin Berlin, Department of Hepatology & Gastroenterology, Campus Virchow-Klinikum and Campus Charité Mitte, Berlin, Germany

**Keywords:** Cholangiocytes, liver fibrosis, liver cancer, NAFLD, MASLD, MASH, PSC, PBC, Immunology, Cell biology

## Abstract

The liver is a central hub in lipid, carbohydrate and protein metabolism and protects against gut-derived antigens and toxins. The etiology of liver diseases includes altered metabolism, viral infections, autoimmunity, toxins and genetic alterations. Liver-resident cells, including hepatocytes, biliary epithelial cells, endothelial cells, and hepatic stellate cells, are essential for liver function and homeostasis but may also drive the development of inflammation, fibrosis, cirrhosis and liver cancer via interactions with immune cells. This review highlights the often-underappreciated contributions of epithelial, endothelial and mesenchymal liver cells in regulating inflammation and immunity across various liver diseases, emphasizing their importance in disease onset, progression and regression. Immune cells and their mediators also play a role in stimulating liver regeneration and repair following injury. Recent findings on the bidirectional interactions between immune cells and resident liver cells provide deeper insights into the underlying pathophysiology and identify novel therapeutic targets for the treatment of liver disease.

## Introduction

### Foreword

This review highlights the inflammation-related pathogenic mechanisms involved in liver disease initiation and progression from a nonimmune cell perspective. This manuscript reviews recent findings on the immune-modulatory functions of hepatocytes, cholangiocytes or biliary epithelial cells (BECs), hepatic stellate cells (HSCs), and liver endothelial cells (LECs). First, we briefly describe central molecular pathways that drive liver organogenesis but can also be reactivated upon liver injury to stimulate organ repair or, if injury persists, may promote disease progression. Next, we elaborate on the known roles of liver epithelial and stromal cells in modulating disease-driving and inflammation-related cellular responses. Finally, we discuss recent state-of-the-art technologies that will further improve our understanding of how interactions between parenchymal and nonparenchymal cells regulate inflammation and immunity in the liver and hold promise for tackling liver disease from novel angles.

### Liver development

The intricate cellular crosstalk networks in the liver take root during embryogenesis, and some of the molecular pathways involved in organogenesis have been found to be reactivated upon liver disease onset, wound repair, and cancer development. Liver development is initiated from the foregut endoderm, which is part of the embryonic digestive tube. Signals such as fibroblast growth factors (FGFs) and bone morphogenetic proteins (BMPs), derived from the cardiac mesoderm and the septum transversum mesenchyme, initiate liver development [1–4]. In addition, the anterior foregut endoderm secretes wingless-related integration site (Wnt) antagonists to inhibit Wnt signaling, allowing for hepatic specification [5]. As the liver grows, cells differentiate into hepatoblasts, which are precursors of both hepatocytes and cholangiocytes or biliary epithelial cells (BECs). By default, hepatoblasts engage in a differentiation path toward hepatocytes. In the portal area, mesenchymal cell-derived signals drive hepatoblasts toward the biliary lineage in mice [6, 7].

During embryonic stages, primitive vascular structures and sinusoids along with early bile ducts guide hepatic cell migration in the liver [8], suggesting a crucial role for endothelial cells during liver development. Indeed, angiocrine signals control liver embryonic growth between E11.5 and E13.5 through the regulation of the Wnt and Notch pathways [9, 10]. During prenatal development, the hemogenic endothelium gives rise not only to endothelial cells but also to hematopoietic stem cells, which seed the fetal liver and contribute to erythro-myeloid hematopoiesis during development and early postnatal stages [11]. The niche that maintains hematopoietic stem cells in the fetal liver is largely supported by stem cell factor (SCF), which is expressed by endothelial cells and hepatic stellate cells [12]. Deletion of SCF in both endothelial cells and hepatic stellate cells leads to nearly complete loss of hematopoietic cells and early lethality in mice [12]. Some mechanisms involved in the close interactions between endothelial cells and hepatic stellate cells during development may also affect these interactions during disease. During postnatal development in mice, there is a distinct subcluster of Kupffer cell-derived *Decorin* (*Dcn*)^+^ macrophages that express liver EC markers such as *Cd31*, *Kdr* and *Lyve1*, as suggested by a recent single-cell RNA sequencing (scRNA-seq) study [13]. These *Dcn*^+^ macrophages closely interact with liver sinusoidal endothelial cells (LSECs) through several VEGF-related receptor/ligand pairs and with Tregs through CXCL12-CXCR3/CXCR4 signaling, as shown by CellPhoneDB [13], suggesting a role for endothelial cell/immune cell crosstalk during postnatal liver development.

### The cellular landscape of the healthy adult liver

**Fig. 1:**
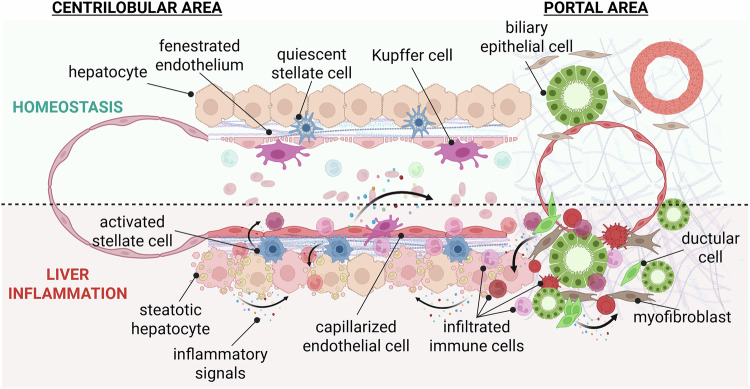
Schematic view of liver histology in homeostasis and upon liver inflammation. The liver microenvironment undergoes drastic alterations upon liver inflammation, which are driven by intricate cellular crosstalk pathways, including the release of damage- and stress-induced inflammatory cytokines or molecular patterns by liver epithelial, stromal, and mesenchymal cells. This figure displays a schematic representation of the cellular organization along the porto-central axis during homeostasis and upon steatotic liver disease, as an example of drastic alterations in the liver cell landscape upon tissue inflammation

Hepatocytes represent approximately 80% of the liver mass and 60% of all liver cells and are organized as a monolayer of cells along the portal‒centrilobular vein axis. Hepatocytes are classically regarded as the key epithelial cell type in the liver and are responsible for most liver functions, including glucose, lipid, protein, and bile acid metabolism as well as detoxification [14]. Biliary epithelial cells, often also termed cholangiocytes, account for ~3–5% of all liver cells. They line the bile ducts and are mostly studied for their roles in transporting and regulating the composition of bile, which is produced by hepatocytes, stored in the gallbladder, and released into the intestinal lumen [15]. Liver sinusoidal endothelial cells (LSECs) form a fenestrated endothelium that lines the liver blood sinusoids and allows for the circulation of solutes and particles between the vascular compartment and the hepatocyte layers. HSCs are a quiescent mesenchymal cell population in the healthy liver and are best known for their function in most of the body’s vitamin A storage in the form of retinyl esters in their characteristic lipid droplets [16, 17]. Increasing evidence points toward important roles of LSECs and HSCs in the regulation of hepatocyte zonation and metabolic functions via the WNT pathway [18, 19]. The healthy liver also contains Kupffer cells as a large resident immune cell population [20]. Since two-thirds of the hepatic supply is via the portal vein, the liver is constantly exposed to gut-derived nutrients, bacteria, and bacterial components. Kupffer cells act as a firewall against bacterial infection [21] and clear blood from lipopolysaccharides (LPS) [22]. In addition to Kupffer cells, hepatocytes, LSECs, and HSCs also possess important immune-sensing capacities via their expression of Toll-like receptors [23, 24], and their response potential to environmental cues contributes to the hepatic response to pathogenic stimuli. The diseased liver is characterized by profound changes in epithelial, mesenchymal, and stromal compartments and is accompanied by a strong accumulation of immune cells (Fig. 1) [25, 26].

## Interactions of epithelial liver cells with immune cells in liver disease

### Hepatocytes

Hepatocytes are mature epithelial cells responsible for most synthetic functions of the liver; these cells have a wide range of systemic effects and affect organismal health. Accordingly, hepatocyte injury and stress can contribute to both local and systemic inflammation through the release of damage-associated molecular patterns (DAMPs), cytokines, and acute phase proteins (APPs). The involvement of these proteins in a wide range of metabolic processes as well as the receptor systems and enzymes needed to import, export, and metabolize substrates and toxins renders hepatocytes a common “victim” in liver injuries caused by a wide range of etiologies, including metabolic, toxic, biliary, and viral liver diseases. Owing to their central position in the organ’s functions and architecture, hepatocytes are fundamentally interconnected with surrounding cells. Following liver injury, hepatocytes, together with surrounding cells, exert a profound influence on inflammatory and immune responses and change and often overcome the normally immunotolerant hepatic environment. Accordingly, hepatocyte–immune interactions actively participate in virtually all steps of liver diseases, from initial hepatitis to progression toward fibrosis, cirrhosis, and cancer. Conversely, immune‒hepatocyte interactions aid in liver regeneration and the restoration of liver architecture after liver injury [27].

#### Hepatocytes drive systemic and local immune responses via acute-phase proteins

The liver plays a central role in the regulation of immune defenses during systemic infections, with roles in bacterial sensing and clearance, APP and cytokine production, and metabolic adaptation to inflammation [28]. Following infection or sterile injury, the liver releases up to 30 APPs into the systemic circulation [28, 29]. The APP response involves the crosstalk of multiple liver cell types with a central role in hepatocytes. Monocytes and macrophages, which sense and trap the majority of bacteria and bacterial pathogen-associated molecular patterns (PAMPs), trigger the production of APPs in hepatocytes via inflammatory cytokines such as interleukin (IL)-1 and IL-6 and the downstream mediators NF-κB and STAT3 [28–31]. Consistent with their function as the primary synthetic cell type in the liver, hepatocytes are the predominant producers of major APPs, including C-reactive protein (CRP), serum amyloid A (SAA), serum amyloid P, complement factors, ferritin, and lipopolysaccharide binding protein [28, 32]. Most of these hepatocyte-produced APPs are key regulators of systemic inflammation and immunity [33] but can also act locally, thereby affecting the development of liver disease. Via APPs, hepatocytes directly contribute to extracellular pathogen clearance via complement-mediated antigen opsonization [34–36]. Importantly, complement cascade activation leads to potent immune responses, and when inappropriate, this can have severe consequences for the organism [37]. Notably, recent studies have investigated the implications of the complement system in liver diseases. C3 complement protein is increased in human metabolic dysfunction–associated steatohepatitis (MASH) livers, and the activation of the alternative complement pathway is associated with disease hallmarks [38]. In addition, C5a levels are increased in patients with liver ischemia after transplantation, but its potential role in ischemia‒reperfusion injury and liver transplant rejection remains under debate [39–42]. Moreover, C5 inhibition is protective in mouse models of acute liver failure, presumably by reducing proinflammatory immune cell recruitment and thrombosis [43]. Similarly, C3 inhibition or a complement receptor CD59 inhibitor improved ischemia‒reperfusion injury, and CD59 inhibition also drastically improved liver regeneration and survival after 90% partial hepatectomy in mice, along with increased TNF-α and IL-6 levels [44]. SAA proteins constitute another group of APPs with key roles in host defense and inflammation [34]. Its two human isoforms, SAA1 and SAA2, are predominantly produced by hepatocytes and contribute to systemic immune responses in sepsis but also in a range of other inflammatory conditions, such as arthritis [34, 45]. In liver injury induced by acetaminophen, SAA1 and SAA2 increase hepatocyte and LSEC damage, thus potentiating acetaminophen-induced microvascular dysfunction [46]. SAA1 may also increase concanavalin-A-induced liver inflammation and injury via a Toll-like receptor (TLR)2-dependent pathway by potentiating Th17 lymphocyte and monocyte activation [47]. The roles of APPs remain diverse and can also drive anti-inflammatory immune responses. As such, the secretion of SAA1 and SAA2 by hepatocytes inhibits dendritic cell differentiation and thus limits T-cell activation and infiltration in extrahepatic cancers [48]. Accordingly, pancreatic adenocarcinoma patients with lower levels of SAA have higher circulating T-cell counts and longer survival after surgery [48]. Similarly, the IL-6/STAT3 pathway leading to SAA production drives the establishment of a prometastatic niche in the liver [49]. Moreover, together with CXCL1, SAA contributes to the recruitment of anti-inflammatory CD11b^+^Gr1^+^ myeloid-derived suppressor cells (MDSCs) in a mouse model of polymicrobial sepsis, leading to innate immune response control and increased survival via the gp130–STAT3 signaling pathway [50]. CRP and amyloid P are APPs of the pentraxin family that are predominantly secreted by hepatocytes and increase 1000-fold following infection or injury [35, 36]. CRP primarily binds to phosphocholine (present on the surface of bacteria and necrotic and apoptotic cells) and C1, driving complement activation, immune responses, and the removal of dead cells [35, 36]. The fact that deletions of CRP have not been reported in patients suggests an essential role in humans [36]. It has been suggested that the ability of CRP and other pentraxins, such as serum amyloid P, to aid in the removal of dead cells may prevent autoimmunity [35, 51]. C-reactive protein (CRP) attenuates complement-driven liver injury in CCl_4_- and acetaminophen-induced liver injury models [52, 53]. Similarly, serum amyloid P has been shown to reduce CCl_4_-induced liver injury and the development of metabolic dysfunction-associated steatotic liver disease (MASLD) in mice [54, 55]. High serum concentrations of lipopolysaccharide binding protein (LBP) are associated with decreased survival in patients with decompensated cirrhosis, and *Lbp* knockdown increases liver inflammation in mice fed a methionine- and choline-deficient (MCD) diet [56, 57]. Alpha-1-acid glycoprotein (AGP) or orosomucoid 1 (ORM1) is another long-known APP secreted by hepatocytes, and its serum levels increase drastically in a number of systemic or extrahepatic inflammatory disorders, with diverse and sometimes opposing effects on immune cells [58, 59]. Its variant, ORM2, has recently attracted significant interest because its decrease is associated with poor prognosis in patients with HCC and metastasis [60, 61]. Similarly, ORM2 levels in the livers and plasma of patients with MASLD as well as in MASH model mice are lower than those in the corresponding controls [62, 63]. In those studies, ORM2 was shown to protect hepatocytes from steatosis while increasing portal inflammation in murine in vivo and in vitro models. Taken together, the secretion of APPs by hepatocytes illustrates the central role of the liver in controlling systemic inflammation and immunity, with potent anti- and proinflammatory effects depending on the disease- and time-specific context. In summary, while most effects of hepatocyte-secreted APPs are systemic, facilitating the clearance of infections and cellular debris after sterile injury, they may also play a role in liver diseases.

#### Immune cell mediators stimulate hepatocyte proliferation and liver regeneration following liver injury

While immune cell‒hepatocyte interactions contribute to the pathogenesis of liver injury and disease, they also drive the restoration of liver mass and function after injury ceases. Many immune‒hepatocyte interactions are likely involved in the elimination of infected, pathogenic, stressed, or dying hepatocytes while promoting the expansion of surviving healthy hepatocytes. These regeneration-promoting effects have been most clearly shown in models of liver regeneration following partial hepatectomy. Kupffer cells and bone marrow-derived macrophages and their mediators, such as TNF and IL-6, play key roles in liver regeneration, as demonstrated by knockout, depletion, and bone marrow transplantation studies [64–70]. Crosstalk between hepatocytes and macrophages, involving metabolic reprogramming of bone marrow-derived macrophages, promotes hepatocyte proliferation in a WNT3–YAP-dependent manner [71]. In addition to Kupffer cells and bone marrow-derived macrophages, peritoneal macrophages can also contribute to the removal of necrotic cells and tissue repair following heat- or CCl_4_-induced liver injury, which delays wound healing after macrophage depletion [72]. The absence of NK and NKT cells but not NK cells alone reduces liver regeneration [73]. Moreover, the activation of NK cells by TLR3 ligands such as poly I:C may also delay liver regeneration [74]. Furthermore, γδT cells promote liver regeneration via IL-17A [75]. Additional immune cell types, such as dendritic cells and innate lymphoid cells, may also contribute to liver regeneration [27].

#### Stressed hepatocytes drive steatotic liver disease-induced inflammation

As a central regulator of lipid, glucose, and protein metabolism, the liver is highly susceptible to changes in energy intake and body composition. Accordingly, obesity and diabetes are closely associated with the development of metabolic dysfunction-associated steatotic liver disease (MASLD). The increasing incidence of MASLD points toward the urgent need for a better understanding of the implications of dysregulated metabolism and lipotoxicity [76, 77]. In the liver, excess lipids lead to the development of steatosis and oxidative stress [77]. Ultimately, chronic hepatocyte injury and cell death lead to the release of DAMPs that trigger liver inflammation or carcinogenic pathways in the liver [78]. These signals directly initiate and potentiate an immune response, which is often regarded as a detrimental actor in liver diseases. A landmark study recently reported the presence of CXCR6^+^ CD8^+^ T lymphocytes, which are autoaggressive via a mechanism distinct from antigen-driven cytotoxicity, in MASH mice and patient samples, thus providing further insights into disease-driving mechanisms [79]. Interestingly, efferocytosis of hepatocyte-derived debris by SIRPα^+^ liver macrophages (both KC-derived and monocyte-derived) represses the onset of liver fibrosis [80, 81]. This mechanism of immune response regulation is further enhanced when the expression of the hepatocyte “don’t eat me” molecule CD47 is blocked with a specific IgG, which also leads to a downregulation of fibrosis-related gene expression and collagen deposition in a mouse model of MASLD [80]. In the present study, mice fed the HF-CDAA diet for 6 weeks and treated with an anti-SIRPα antibody for another 6 weeks presented increased efferocytosis of hepatocyte-derived necrotic bodies, decreased collagen deposition, and a reduced α-SMA^+^ area and Opn^+^ stained areas.

In patients with chronic alcohol-associated liver disease (ALD), acute-on-chronic severe liver injury or alcohol-associated hepatitis (AH) may occur in rare cases. In ALD, hepatocyte cell death results in the release of DAMPs that, together with gut-derived PAMPs, activate surrounding immune cells and serve as recruiting signals for circulating immune cells [82, 83]. In addition, in mouse models of ALD and patients with AH, hepatocytes overexpress macrophage migration inhibitory factor (MIF), and higher levels of MIF in patient suprahepatic serum are associated with increased markers of liver injury and slightly yet significantly increased mortality [84]. The authors suggested that this detrimental role of MIF may be related to its chemotactic functions. Neutrophils are also potent inducers of hepatocyte injury in AH through the release of reactive oxygen species (ROS) [85] and via degranulation [86]. Similarly, higher levels of hepatic IL-8 and CXCL5 expression are associated with poor survival in AH patients [87]. However, recent data suggest that IL-8 is produced mainly by neutrophils in AH [88]. Moreover, the hepatocyte endoplasmic reticulum (ER) stress-related transcription factor ATF4 leads to increased LECT2 expression in neutrophils, which is key in neutrophil-induced liver injury and neutrophil extracellular trap (NET) formation in ALD [89]. However, it has also been suggested that neutrophils can be restorative and promote liver regeneration, and the disease-promoting versus pathogenic roles of neutrophils [90, 91] are likely time-, context- or subpopulation-dependent.

#### Antigen and lipid presentation by hepatocytes drive immune cell activation

Compared with other organs, the liver is characterized by an immunotolerant environment, highlighted by a high rate of chronic viral infections [92] as well as successful transplantation despite major histocompatibility complex (MHC) mismatch [93]. Although hepatocytes can present antigens to T cells through MHC-I [94, 95] and trigger antiviral immune responses after murine hepatitis virus B infection [94, 96, 97], the efficacy of local priming in the liver for virus-specific CD8 + T-cell immunity is low, often leading to dysfunctional CD8 + T cells [98, 99]. This may be due to the absence of survival signals such as IL-2 [98, 100], Bim-mediated lymphocyte death [101], and the ability of hepatocytes to eliminate CD8 + T cells by engulfment in a process termed suicidal emperipolesis [94, 99]. Suicidal emperipolesis has been investigated primarily in the context of autoreactive T cells in autoimmune hepatitis but may also contribute to tolerogenic environments in other settings, such as viral hepatitis [94, 99]. Efficient antiviral immune responses may require priming by Kupffer cells and/or secondary lymphoid tissues by professional antigen-presenting cells (APCs) rather than hepatocytes and subsequent migration of cytotoxic effector CD8^+^ T cells to the liver [98, 99, 102]. While some infections with hepatotropic viruses, such as HCV, often become chronic, others can be efficiently cleared, such as infection with HBV in adulthood [103]. In addition to the induction of efficient CD8^+^ T-cell responses via professional APC priming, viral clearance is aided by the high sensitivity of infected hepatocytes to TNF-dependent CD8^+^ T-cell-induced cell death and allows the clearing of large numbers of infected hepatocytes by a relatively small number of CD8^+^ T cells (outnumbered 100--1000) [99, 104]. Hepatocytes also present lipid antigens via CD1d, which leads to the activation of natural killer T (NKT) cells in the context of HBV infection [105]. The absence of NKT cells, CD1d, or a defective transfer of lipids onto CD1d leads to diminished HBV-specific T and B-cell responses and delayed viral control [105]. In some settings, such as infection with the parasite *Schistosoma japonicum*, hepatocyte CD1d is downregulated, which may exacerbate liver disease [106]. Accordingly, AAV8-mediated Cd1d overexpression results in decreased NKT cell numbers; downregulation of IL-13, IL-4, and IFN-gamma; and reduced liver damage in this model [106]. Moreover, hepatocytes aberrantly express the major histocompatibility class II (MHC**-**II) transactivator, known as CIITA, when exposed to IFN-gamma and during hepatitis B virus infection as part of a protection mechanism against HBV replication [107, 108]. Hepatocytic MHC-II expression has also been detected in alcohol-associated hepatitis (AH), primary sclerosing cholangitis (PSC), and AIH patient samples [109, 110]. However, this unexpected MHC-II expression by hepatocytes has only been described in a few studies and needs further robust validation [111]. Ultimately, characterizing the peptides presented by hepatocytes and leading to an immune response holds promise for patients affected by diseases such as viral hepatitis or hepatocellular carcinoma [106]. Hepatocytes are equipped with many innate immune sensors, which represent the first line of defense against any pathogen [92]. Innate immunity is largely based on pattern recognition receptors (PRRs) [112]. Hepatocytes functionally express a wide range of PRRs that recognize pathogen-associated molecular patterns from viruses and bacteria in different cellular compartments. These include RIG-I-like receptors (RLRs), retinoic acid inducible gene-I (RIG-I), melanoma differentiation antigen 5 (MDA5), Nod-like receptors (NLRs), and Toll-like receptors [92, 113]. PRR activation leads to inflammatory and antiviral responses, particularly the expression of interferons and hundreds of interferon-stimulated genes [92]. Moreover, it drives the recruitment of adaptive immune cells and enhances adaptive immune responses [114]. While these responses are effective for many viral and bacterial infections, hepatitis C virus (HCV) often causes chronic infections. Although hepatocytes sense PAMPs from HCV and mount an initial innate immune response, HCV proteins such as NS3/4a can inhibit innate immune responses by proteolytically targeting MAVS (essential for RIG-I signaling) and TRIF (essential for TLR3 signaling) [92]. The resulting moderate activation of PRRs and innate immunity is associated with viral persistence [92]. Accordingly, a high dose of interferon alpha can lead to HCV clearance in acute or chronic HCV infection [115]. Hepatocytes also express TLRs, including TLR4, that detect bacterial PAMPs such as LPS. Using hepatocyte-specific knockout approaches, TLR4 on hepatocytes has been shown to trigger innate immune responses that contribute to MASLD and MASLD-induced liver fibrosis [116, 117]. While TLR4 signaling is implicated in the development of alcohol-related liver disease and other forms of liver fibrosis [23, 118], the specific role of hepatocyte-expressed TLR4 needs to be further investigated in these diseases.

#### Hepatocytes influence immune cell activation profiles in cholestatic liver disease via bile acid-derived signals

Hepatocytes represent the primary source of bile acids, which are involved in numerous interorgan and cell‒cell crosstalk pathways as well as immunity and are toxic to hepatocytes and many other cell types [119]. Hepatocyte-specific deletion of *Fxr*, the main receptor for bile acids, results in delayed hepatocyte proliferation following partial hepatectomy [120]. FXR has been termed a “guardian” of hepatic function, as one of its main functions is to lower the levels of bile acids by converting them into cholesterol and secretion into bile [121]. The absence of FXR leads to increased bile acid levels and the development of hepatocellular carcinoma [122]. Moreover, the interruption of bile acid reuptake by hepatocytes via pharmacological NTCP blockage alleviates acetaminophen-induced acute liver injury [123]. High bile acid levels can indirectly trigger inflammation and immune cell recruitment [124, 125]. For example, cholic acid (CA)-treated HepG2 cells generate proinflammatory signals through increased RIPK3 activity, leading to cell death and IL-8 release [126]. Defects in the bile salt export pump Bsep reduce LPS clearance in mice, suggesting the importance of bacterial compound elimination through the bile [127]. Recently, a study in cholestatic mice with hepatocyte-specific *Stard1* deletion demonstrated that STARD1 is key in sustaining excessive bile acid synthesis by preventing the reduction in CYP27A1-mediated bile acid synthesis via the mitochondrial pathway, thereby sensitizing hepatocytes to cytotoxicity and ultimately potentiating liver inflammation [128]. Runt-related transcription factor-1 (RUNX1) levels are increased in mice and patients with cholestasis, and liver-specific *Runx1*-deficient mice subjected to bile duct ligation as well as mouse and human cell cultures revealed that RUNX1 is involved in bile acid-induced *Ccl2* and *Cxcl2* expression by hepatocytes through a signaling pathway that remains to be identified [129]. In addition to these proinflammatory effects, bile acids also exert anti-inflammatory effects mediated by FXR- and TGR5-mediated inhibition of the NLPR3 inflammasome [130–132].

In addition to indirectly modulating inflammation and immune cell recruitment (reviewed in [124, 125]), bile acids also direct immune cell activation profiles via specific receptors such as TGR5 (encoded by *GBAR1*) and FXR [130]. TGR5 and FXR are expressed by Kupffer cells, hepatic bone marrow-derived macrophages, dendritic cells, and NKT cells [130]. However, data from mice with conditional knockout of TGR5 and FXR in immune cells revealed increased inflammation, suggesting that these bile acid-activated receptors have anti-inflammatory effects on the immune system [130, 133, 134]. Moreover, secondary bile acids such as 3β-hydroxydeoxycholic acid have been shown to act directly on DCs to diminish their immunostimulatory properties and increase the differentiation of T regulatory cells [135]. In summary, the predominant direct effects of bile acids on the immune compartment appear to be suppressive.

#### Hepatocytes participate in shaping the immune microenvironment in liver cancer

Primary liver cancers are the 3^rd^ leading cause of cancer-related deaths worldwide [136]. Owing to the high mortality rate, there is a critical need for a better understanding of the pathomechanisms leading to cancer initiation and progression. Current trends show a transition from chronic viral hepatitis to metabolic and alcohol-related liver cancers in many countries. Hepatocellular carcinoma (HCC) represents approximately 90% of primary liver cancers [137]. A vicious cycle of chronic inflammation, injury and inflammation, present in chronic liver diseases such as viral hepatitis, alcohol use disorders, or MASLD, is a key driver of hepatocarcinogenesis, contributing to the accumulation of mutations in hepatocytes and their transformation [138]. Studies in mice have demonstrated that chronic inflammation, e.g., driven by the overexpression of lymphotoxin in hepatocytes [139], and chronic cell death, e.g., by the deletion of MCL-1, NEMO or TAK1, are sufficient to trigger HCC development [140–142]. While chronic inflammation can contribute to hepatocarcinogenesis, the immune system also plays a key role in restraining the development of HCC by removing senescent and transformed hepatocytes and cancer cells [143–145]. Numerous checkpoint proteins, including PD-1/PD-L1, CTLA4, LAG3, and TIM3, are involved in the tumor-immune synapse and the modulation of antitumor immune responses in HCC [144]. Checkpoint molecules such as PD-L1, which are expressed by tumor-associated macrophages and cancer cells, bind to PD-1, which is expressed by T cells, and deactivate it, thus preventing antitumor adaptive immune responses [146]. Accordingly, combinations that include checkpoint inhibitors targeting PD-1/PD-L1 and CTLA4 represent current first-line therapies for HCC [144]. Growth differentiation factor-15 (GDF-15) represents another, largely hepatocyte- and tumor cell-expressed cytokine that is increased in MASLD [147] and suppresses antitumor immunity [148]. The anti-GDF-15 antibody visugromab has been evaluated in combination with anti-PD-1 therapy in patients with anti-PD-1/PD-L1 refractory tumors [148]. Erythropoietin (EPO) represents another tumor cell-derived mediator that suppresses antitumor T-cell responses in HCC by interacting with the EPO receptor on tumor-associated macrophages [149]. Accordingly, HCC patients with high expression of EPO have worse survival [149]. It is likely that additional hepatocyte- and tumor cell-expressed mediators contribute to a changed immune environment and the suppression of immunity in injured and tumor-bearing livers, promoting hepatocarcinogenesis or tumor progression, respectively.

#### Hepatocytes as immune cells that target autoimmune hepatitis

Autoimmune hepatitis (AIH) is a chronic liver disease primarily characterized by the detection of autoantibodies, progressive hepatocellular necrosis and inflammation, leading to liver fibrosis and, ultimately, cirrhosis. Its prevalence is three times higher in females than in males [150, 151]. The immune mechanisms leading to disease progression have been studied in detail and mostly rely on the adaptive immune response against hepatocyte-expressed antigens and defects in tolerogenic pathways [152, 153]. Recent insights have further highlighted the implications of B cells in amplifying disease-driving pathogenic immune responses [154]. The initial causes and impacts of environmental cues remain to be determined, although genetic factors have been identified in some cases [150].

### Biliary epithelial cells

Biliary epithelial cells (BECs) and their derivatives, often referred to as ductular cells in liver diseases, play a key role in many liver diseases, as the transport of bile to the intestine is a central liver function that needs to be preserved to avoid toxic effects on the liver. Moreover, their microanatomic niche predisposes BECs to interact with many other cell types, including immune cells, and renders the periductular area a common site of inflammation [155–157]. Earlier studies revealed developmental and functional differences between intra- and extrahepatic BECs and between large and small BECs [15]. Moreover, BECs and ductular reactions have sparked interest as potential reservoirs of alternative liver progenitor cells, a phenomenon that also relies on the participation of innate and adaptive immune cells [158–160]. Similarly, more recent findings further elaborate on the hepatocyte-cholangiocyte phenotypical plasticity that takes place during liver regeneration [161]. Furthermore, an increasing amount of data highlights BECs as both targets and active potentiators of immune responses in the liver. Recent technological developments, such as single-cell transcriptomic and proteomic analyses along with novel algorithms to explore complex data, have provided deeper insights into rarer cell populations, such as BECs, in the context of animal models and human biliary diseases [162–166]. Monocytes and macrophages are key partners of reactive BECs, as exemplified by studies indicating that macrophage-derived TWEAK is one of the initiating signals for ductular cell proliferation via the Fn14 receptor [167, 168]. This privileged cellular crosstalk is supplemented by potent interactions between BECs and, notably, B lymphocytes and monocytes-macrophages, as detailed below.

#### There is interest in understanding the inflammatory mechanisms involved in cholangiopathies

As the archetype of BEC-centered pathologies, cholangiopathies are of distinct genetic, environmental, or idiopathic nature. Cholangiopathies include primary biliary cholangitis (PBC), primary sclerosing cholangitis (PSC), liver cystic fibrosis, biliary atresia, and cholangiocarcinoma. Much remains to be discovered concerning the mechanisms driving both the initiation and progression of these relatively rare conditions. Nonetheless, in the past decade, substantial progress has been made in both understanding cholangiocyte biology and its role in liver diseases [169]. Recently, in an elegant spatially resolved transcriptome analysis of a mouse model of ductular reaction (3,5-diethoxycarbonyl-1,4-dihydrocollidine, or DDC diet), during injury, cholangiocytes represented a major cell crosstalk hub in the portal area through predicted interactions with virtually all immune and parenchymal cells in their surroundings [170]. Cholangiocytes are particularly active in expressing chemoattraction-related genes and are suggested to inhibit hepatocyte-driven liver regeneration through TGF-β2 and Atoh8 expression in the DDC model. Interestingly, this study also revealed that increased cholangiocyte stress may be attributed to a decrease in the cholangiocyte bicarbonate umbrella, which results in increased sensitivity of biliary cells to bile acids [170]. Indeed, one of the main functions of BECs lining the bile ducts is to allow for the transport of bile from the liver to the intestines. Thus, alterations in the biliary compartment may lead to either a lack of bile efflux from the liver or leakage from the bile ducts, leading to both BEC and hepatocyte injuries and liver inflammation as a consequence of toxic bile exposure. During homeostasis, BECs are protected from bile toxicity through the bicarbonate umbrella. The presence of anti-annexin A11 IgG1/IgG4 autoantibodies drives IgG4-related cholangitis pathogenesis by altering this protective HCO3− secretion [171].

The pathogenesis of PSC remains unknown, and consequently, no effective therapy has been identified at the time this review was written, although ongoing studies are exploring potential candidates, including UDCA [172]. PSC is strongly associated with inflammatory bowel disease (IBD), although the causes remain to be characterized [173]. However, PSCs exhibit some signs of an autoimmune disease directed against BECs, and evidence points toward intense crosstalk between BECs, neutrophils, macrophages, and T and B cells [174, 175]. PSC patients are usually identified at later disease stages, making identifying the disease-causing mechanisms challenging. Finally, PSC is considered a risk factor for intrahepatic cholangiocarcinoma [176]. Recently, endoscopic retrograde cholangiopancreatography (ERCP)-derived extrahepatic cholangiocyte organoids generated from PSCs and non-PSC patients were shown to be largely similar when cultured in vitro, notably in terms of their BEC marker expression and BEC landmark functions, and they displayed similar responses to inflammatory cytokine stimulation [176]. While these results open possibilities for autologous cell-based therapies in PSCs, they also propose that the causes of PSC, which remain to be identified, are not due to intrinsic changes within BECs but may be the consequences of changes within the BEC microenvironment in the liver.

PBC may represent the best-characterized type of cholangiopathy, although some characteristics, such as a female preponderance, remain to be explained. PBC is an autoimmune disease that primarily involves the targeting of intrahepatic BECs and results in chronic liver inflammation and fibrosis, in which hepatocyte injury is secondary and only manifests in later stages due to increased inflammation and cholestasis leading to oxidative stress [177]. Gene variants in HLA or IL-12 signaling have been associated with PBC, and most patients display autoreactive antibodies against mitochondrial antigens and subsequent failure of biliary transporters, e.g., anion exchange protein 2 (AE2) [177–179]. However, immunosuppression alone is insufficient for treating PBC patients [180]. Instead, ursodeoxycholic acid (UDCA), a hydrophilic bile acid, is used as a first-line therapy for PBC to reduce bile acid toxicity and, thus, liver inflammation and hepatocyte injury [177]. Interestingly, single-cell sequencing data acquired from PBC patients revealed a decreased bicarbonate umbrella and metabolism-related gene expression and increased chemokine, coagulation, and phagosome-related gene expression in cholangiocytes [181]. Importantly, variants in the 19p13.3 locus were shown to play a role in PBC pathogenesis in two Chinese cohorts through increased autophagy-related *ARID3A* enhancer activity in CD14^+^ monocytes [182]. This further points toward landmark monocyte accumulation near BECs in PBC progression, which may represent a yet unexplored target for drug development, an observation made by us and others in PBC patient liver samples [175]. Recently, live CD8^+^ CD69^+^ CD103^+^ E-cadherin^high^ T cells were shown to be internalized by, or invade, BECs in PBC patient samples and to a lesser extent in healthy livers [183]. However, the functional implications of CD8^+^ T-cell invasion in BECs remain to be clarified.

#### BECs and B cells: evidence for a disease-driving mechanism in autoimmune cholangiopathies

Biliary atresia is a cholangiopathy that affects infants and is characterized by intra- and extrahepatic bile duct paucity, leading to bile build-up in the liver and jaundice, for which the primary treatment remains surgery with the Kasai procedure to allow bile flow to the intestines [184]. Circulating CXCL8/IL-8-expressing B cells, which are considered proinflammatory, are found in the peripheral blood of biliary atresia patients [185]. In addition, a rhesus rotavirus (RRV)-induced neonatal mouse model of biliary atresia leads to liver infiltration by B cells, and B-cell deficient mice are resistant to bile duct injury [186]. Interestingly, the authors demonstrated that B-cell-mediated bile duct injury, as well as macrophage and T-cell activation, are independent of the antigen presented by B cells in this model. B-cell infiltration and high levels of autoantibodies are also evident in *Mdr2*^−/−^ mice, and B-cell depletion by an anti-CD20 antibody leads to reduced fibrosis and inflammation [187]. Liver-infiltrating antibody-secreting B cells are also detected in both PSCs and PBC, although fewer IgM-positive plasma cells are observed in PSCs than in PBC [188]. On the other hand, IgA autoantibodies have been measured in PSCs independently of their association with intestinal bowel disease (IBD) [189]. However, the association between autoantibodies and PSC remains to be fully elucidated. Recently, a study in a mouse MASH model revealed that intestinal IgA release induces the activation of CD11b^+^CCR2^+^F4/80^+^CD11c^-^FCGR1^+^ liver macrophages through the Fc-receptor gamma [190]. Although ductular reactions have not been investigated in the latter, it is reasonable to propose that the BEC‒macrophage axis is involved in the response to gut inflammation.

#### Gut-derived signals trigger portal area inflammation through BECs

Recent data point toward a direct link between cystic fibrosis transmembrane conductance regulator (CFTR)-induced gut dysbiosis and increased liver injury through ductular reactions, which include portal inflammation in CFTR-deficient mice [191]. BECs are known to express TLRs 1–6, and in particular, human BECs are particularly sensitive to poly:IC (a TLR3 ligand), which leads to their apoptosis through TRAIL [192]. Similarly, TLR2 ligands induce apoptosis in a mouse cholangiocyte cell line, along with increased *Cxcl10*. Interestingly, the inflammatory cytokines TNF-alpha and CCL2, as well as ICAM-1, are increased in BECs in a murine model of autoimmune cholangitis, further driving CD8^+^ T-cell-mediated portal inflammation. In addition, the authors proposed that TNF-alpha increases TLR2 expression on BECs and, thus, their sensitivity to gut microbiota antigen-induced cell death. In addition, microbial antigen sensing is generally attributed to liver-resident macrophages, and proinflammatory TLR signaling is inhibited by triggering receptor expressed on myeloid cells-2 (TREM-2). Accordingly, *Trem2*-deficient mice subjected to bile duct ligation (BDL) developed a more severe phenotype than did their wild-type counterparts, as indicated by a more prominent ductular reaction [193]. TREM-2 expression is positively correlated with both disease progression and macrophage recruitment in PSC and PBC patients and in corresponding mouse models, suggesting that TREM2 overexpression is a mechanism that responds to cholestasis by repressing the proinflammatory activation of liver macrophages, a process that is amplified by UDCA. In addition, TREM-2 overexpression reduces IL-33 expression, and IL-33 has been shown to favor mouse BEC proliferation [194]. Moreover, in vitro LPS stimulation of human BECs leads to the release of T-cell chemokines such as CCL20 and Th17-polarizing cytokines, including IL-1β and IL-6 [195]. IL-17-producing cells accumulate in the periportal area and sustain liver fibrosis while potentiating ductular cell accumulation [159, 196–198]; however, this study further suggests a direct role of BECs in driving this disease-promoting, local recruitment and polarization of Th17 cells [195]. Notably, IL-17A overexpression in the perilobular area induces the transformation of ductular cells into cancer stem cells, suggesting that the Th17-enhancing roles of BECs may be involved in liver cancer initiation [199]. In line with these findings, it is no surprise that ductular cell accumulation is associated with immune cells in a variety of chronic liver diseases. Overall, these studies support the hypothesis that intricate crosstalk between BECs and immune cells is a driving force for the liver ductular reaction and is under the influence of gut-derived signals.

#### Reactive BECs and monocyte–macrophage complexes closely interact in acute liver diseases and in MASLD

As with virtually all (liver) cells, our view of the range of cytokines secreted by BECs continues to expand as new results are released. In reference to “cholangiokines”, the BEC secretome is versatile, and we have just started to explore its potential for shaping portal inflammation [156]. In a mouse model of acute and targeted BEC injury and in the absence of confounders, we found that BECs engage in cell proliferation while expressing genes associated with chemotaxis, including the *Ccl2* chemoattractant for monocytes [200]. This BEC CCL2-driven monocyte recruitment was also observed in mice injected with BV6, another model of acute cholangitis, and in human cholangiocytes exposed to BV6 in vitro [201]. Reciprocally, monocytes can drive a ductular reaction in the absence of additional injury [167]. Moreover, the joint response of monocytes and reactive BECs supports biliary tree repair and, during severe liver injury, supports hepatocyte regeneration [200, 202]. TRAIL knockout in myeloid cells was shown to exacerbate a proinflammatory phenotype in cholangiocytes in a DDC model [203]. This BEC proinflammatory phenotype was particularly marked by increased gene expression of *Cxcl1*, a typical neutrophil chemoattractant, which is associated with increased neutrophil recruitment and liver fibrosis. Notably, neutrophils drive ductular cell expansion and liver fibrosis in the same murine DDC model [204]. In patients, CCL28-expressing macrophages are observed near ductular cells but not close to large bile ducts in PSC livers [205]. Similarly, despite very distinct disease features, higher ductular cell densities have been associated with an increased extent of hepatocyte loss and with the number of monocytes accumulating in inflamed portal areas in MASLD/MASH [175, 206, 207]. Hence, more research is needed to fully decipher the intricate crosstalk between BECs and myeloid immune cells. Similarly, we recently showed in a primary mouse cell-based biliary niche-on-a-chip model that injured BECs release APP ORM2, shifting liver macrophages to a phenotype marked by increased secretion of pro- and anti-inflammatory cytokines [62]. Notably, recent insights from single-nuclei sequencing analyses of patient liver samples suggested that hepatocyte-cholangiocyte cellular plasticity is a key mechanism of liver repair in MASLD [161]. A similar mechanism of cellular plasticity has been demonstrated in mice, where concanavalin-A injections lead JAG1-expressing monocytes to induce the expression of hepatocyte *Sox9*, a typical ductular cell marker, near necrotic lesions. Considering, as mentioned above, that ductular and immune cells are reciprocally supportive, this finding suggests that the BEC-immune cell axis holds high potential for liver regenerative medicine, particularly in MASLD/MASH.

#### Insights into intrahepatic biliary tree-derived malignancies

Patients with PSC, fluke infections, and MASLD have an increased risk of cholangiocarcinoma, whereas patients with PBC have a relatively lower risk [208, 209]. Intrahepatic cholangiocarcinoma (ICC) is a biliary tree neoplasia that arises in noncirrhotic and cirrhotic livers and has a rising incidence [210, 211]. It is thought that the majority of ICCs are derived from BECs [210], but mouse and human hepatocytes also represent potential sources for ICCs [212, 213]. Reduced T and B lymphocyte intrahepatic and increased tumor-associated intrahepatic and CD14^+^CD16^+^ circulating monocyte numbers are associated with poor survival in patients with intrahepatic cholangiocarcinoma [214–217]. Moreover, high PDHA1 succinylation is associated with poor survival, and in mice, CD8^+^ T-cell depletion reversed the protumoral effects of PDHA1 succinylation [218]. In conclusion, much remains to be discovered, but cholangiocarcinoma is a highly desmoplastic and cold tumor with low immune cell infiltration and heterogeneous intratumor areas [219, 220].

**Fig. 2:**
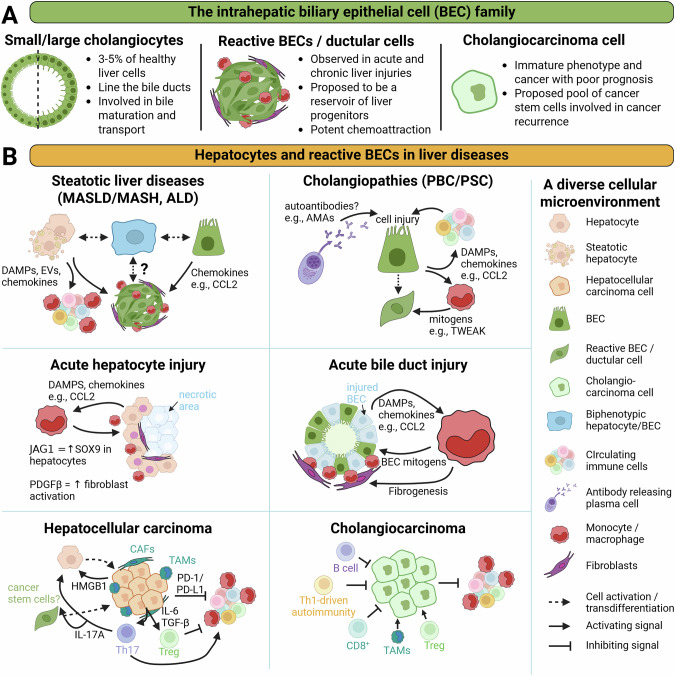
Intrahepatic BEC diversity and implications of BECs and hepatocytes in liver diseases. **A** This schematic view summarizes the diverse forms that BECs can adopt in healthy and diseased livers. **B** Key BEC- and hepatocyte-driven mechanisms regulating liver immune cell recruitment and activation in a variety of etiologies. Abbreviations: ALD: alcohol-related liver disease; AMAs antimitochondrial antibodies, BECs biliary epithelial cells, CAFs cancer-associated fibroblasts, CCL chemokine (C-C motif) ligand, DAMPs damage-associated molecular patterns, EVs extracellular vesicles, HMGB1 high mobility group box 1 protein, ILs interleukins, JAG1 Jagged-1, MASH metabolic dysfunction–associated steatohepatitis, MASLD metabolic dysfunction–associated liver disease, PBC primary biliary cholangitis, PD-1/PD-L1 programmed cell death protein 1/programmed death-ligand 1, PDGFβ platelet-derived growth factor beta, PSC primary sclerosing cholangitis, SOX9 SRY-box transcription factor 9, TAMs tumor-associated macrophages, TGF-β transforming growth factor-beta

Overall, the BEC family is very diverse in its role in liver diseases, and much remains to be understood about the active roles played by ductular reactions in liver disease progression (Fig. 2).

## Hepatic stellate cells and their interactions with immune cells in health and disease

### HSCs—a communication hub and mediator of homeostasis in the healthy liver

**Fig. 3:**
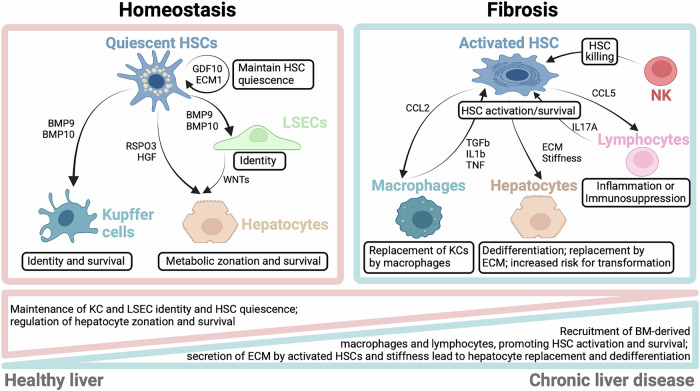
HSC interactions in healthy and diseased livers, switching from homeostasis to inflammation and fibrosis. In the healthy liver, HSCs maintain homeostasis by secreting factors such as BMP9, BMP10, GDF10, ECM1, RSPO3, and HGF. BMP9 and BMP10 are important for Kupffer cell survival and LSEC identification. RSPO3 promotes hepatocyte metabolic zonation. HGF promotes hepatocyte survival. GFD10 and ECM1 maintain HSCs in a quiescent state. In chronic liver disease, these homeostatic HSC factors are progressively lost and replaced by pathogenic mediators driving interactions that keep HSCs in an activated state, promoting fibrosis. HSCs recruit bone marrow-derived monocytes, which then secrete TGF-β, IL-1β and TNF to promote HSC activation and survival. HSCs contribute to the recruitment of lymphocytes via the secretion of chemokines such as CCL5, which in turn maintain HSC activation. The secretion of ECM and the ensuing stiffness result in hepatocyte dedifferentiation, hepatocyte replacement, and an increased risk for transformation into cancer cells. NK cells may limit fibrosis by killing activated HSCs. Abbreviations: BM bone marrow, ECM extracellular matrix, HSC hepatic stellate cell, KC Kupffer cell, LSECs liver sinusoidal endothelial cell, MoMF monocyte-derived macrophage, NK natural killer

HSCs constitute the primary mesenchymal cell population of the liver [221]. They constitute approximately 5-8% of the cells in the healthy liver, where they reside in the space of Disse between the endothelial cells lining the liver sinusoids and hepatocytes [221]. This anatomical position places HSCs in close contact with endothelial cells, hepatocytes, and Kupffer cells as well as other resident and circulating or infiltrating immune cells [222]. Indeed, analyses of ligand‒receptor interactions in single-cell RNA sequencing datasets have revealed that HSCs are among the most interactive cell types in the liver [19, 223, 224]. HSCs not only engage in direct and bidirectional communication with hepatocytes, endothelial cells, macrophages, and immune cells but are also part of multicellular communication networks that control liver functions in health and disease (Fig. 3). For example, HSCs, together with LSECs, regulate the expression of hepatocyte genes, notably those required for the hepatocellular activation of the beta-catenin pathway [19]. Similarly, HSCs provide signals to endothelial cells via GDF-2 and BMP10, which in turn regulate liver macrophage specialization as well as hepatocyte function and zonation [225]. Thus, it appears that some hepatocyte-regulatory circuits in the liver involve multicellular modules with HSCs playing a central role [226]. In the healthy liver, HSCs are in a quiescent state (qHSCs), in which they store large amounts of retinoids in the form of retinyl esters [16, 221]. Nearly 50–80% of the body’s retinoids, which are potent regulators of innate and adaptive immune responses, are stored in HSCs [17]. Moreover, qHSCs are enriched in cytokines and growth factors, including hepatocyte growth factor (HGF), bone morphogenetic proteins (BMPs), and R-spondin 3, and this population has therefore also been termed cytokine- and growth factor-enriched HSCs (cyHSCs) [19, 224, 225]. qHSCs are considered homeostatic because they maintain liver zonation and function via R-spondin 3 [19] and BMPs [225] and protect hepatocytes from injury via HGF [224]. Accordingly, HSC-selective deletion of R-spondin 3 alters liver zonation, metabolism, and regeneration [19]. The HSC-selective deletion of BMP9 and BMP10 alters Kupffer cell and endothelial cell homeostasis [225]. The HSC-selective deletion of HGF predisposes the liver to increased liver injury [224]. Overall, in light of recent studies, HSCs clearly perform diverse functions in the liver under both homeostatic and pathogenic conditions; thus, further studies are needed to understand the role of qHSCs/cyHSCs in the immune homeostasis of the healthy liver.

### Hepatic stellate cells switch from hepatoprotection to fibrogenesis following liver injury

Following injury, HSCs undergo a well-characterized activation process and become the primary fibrogenic cell type of the liver [221, 227, 228]. HSC activation is driven by a wide range of mediators, with TGF-β and PDGF representing the two most potent fibrogenic activators of HSCs [228]. Activated HSCs are best known for producing fibrillar collagens such as type I collagen but also secrete a wide range of other extracellular matrix (ECM) components [221, 228, 229]. Continuous production of ECM by HSCs leads to the development of liver fibrosis. In more advanced disease stages, the ECM can replace functional liver parenchyma and thereby contribute to the development of liver cirrhosis, which is characterized by bridging fibrosis, regenerative nodules, and the loss of liver function [230]. In addition to ECM production, activated HSCs release fibrogenic extracellular vesicles, mainly in ECM-rich zones, to amplify liver fibrosis [231–233]. Activated HSCs also upregulate α-SMA and become contractile through protocadherin 7 (PCDH7) and may thereby contribute to portal hypertension [234, 235]. The increase in these disease-driving interactions is accompanied by a loss of the above-described homeostatic and protective mediators BMP9, BMP10, GDF10, RSPO3, and HGF [226] (Fig. 3).

### Bidirectional interactions between hepatic stellate cells and immune cells

In addition to interacting with endothelial cells and hepatocytes, HSCs also engage in bidirectional interactions with a wide range of immune cells [236, 237]. For example, HSCs regulate Kupffer cell homeostasis via BMP9/10 [225]. Conversely, Kupffer cells are essential for HSC activation and survival via Kupffer cell-secreted TGF-β, TNF-α, and IL-1β [238]. Similarly, HSCs may regulate the recruitment and functions of diverse subsets of immune cells, while immune cells regulate the activation and death of HSCs, as discussed in further detail in the following sections.

### Regulation of HSC death, survival, and activation by immune cells

Macrophages are key mediators of HSC activation, as shown by genetic and pharmacologic depletion [23, 239, 240]. It is believed that the profibrogenic effects of macrophages are largely mediated by TGF-β and that TGF-β derived from activated HSCs may perpetuate HSC activation in more advanced disease stages [221, 228]. Recent studies have suggested that physical interactions between macrophages and fibroblasts are needed to allow for TGF-β-mediated fibroblast activation in a mechanically soft environment [241]. Additionally, ILC2s (via IL-33), T cells (via IL-17), B cells (immunoglobulin independent), Tregs (via amphiregulin) and platelets have been suggested to directly promote HSC activation [197, 237, 242–245]. Moreover, many immune cells promote liver steatosis and MASH and, thereby, indirectly promote HSC activation and liver fibrosis [246]. In addition to the promotion of HSC activation and proliferation by immune cells, HSC survival and death are also regulated by immune cells. Macrophages secrete not only TGF-β to activate HSCs but also TNF-α and IL-1β to promote HSC survival [238] (Fig. 3). Other immune cell subsets, such as NK cells, can induce HSC death in an NKG2D- and TNF-related apoptosis-inducing ligand-dependent manner and, as a result, ameliorate liver fibrosis [247]. These intricate immune cell‒HSC interactions regulate the number of HSCs and thereby allow fine-tuning of wound healing, regeneration, and restoration in different disease stages.

### Antigen presentation and the regulation of immune cell identity and function by HSCs

The liver is known to be a classical immune-privileged organ [248]. Among several cell populations, HSCs have been shown to positively and negatively regulate hepatic immunity. The release of retinoids from HSCs and their subsequent conversion to retinoic acid can maintain regulatory T (Treg) cell differentiation and thereby immune tolerance [249]. Activated HSCs can veto T cells via ICAM1- and contact-dependent mechanisms [250]. Moreover, HSC-expressed PD-L1 may contribute to the inhibition of T-cell- and B-cell-mediated immune responses [251, 252]. Similarly, HSC-expressed TGF-β may inhibit T-cell-mediated immunity [236]. On the other hand, HSCs express MHC class II molecules and have been suggested to serve as antigen-presenting cells (APCs) [253, 254]. However, the antigen-presenting role of HSCs remains controversial, as other studies have shown that HSCs do not cross-present antigens [255]. Furthermore, innate immune receptors on HSCs, such as TLR4 and TLR9, may promote hepatic inflammation [256, 257].

### Hepatic stellate cells and their interactions with immune cells regulate the development and regression of liver fibrosis in chronic liver diseases, including MASLD

In patients with MASLD, liver fibrosis represents the primary determinant of mortality [258–261]. Therefore, the activation process that differentiates HSCs from quiescent and homeostatic cells to ECM-producing fibroblasts represents a key disease-driving process in MASLD [262]. Like in many other liver diseases, the macrophage pool changes in MASLD, with a loss of resident Kupffer cells and an increase in bone marrow-derived macrophages [20, 263]. While the functions of macrophage subpopulations need further clarification, they may play important but distinct roles in HSC activation and fibrosis [264–266] as well as in the repair and resolution of liver fibrosis, e.g., via TREM2 [267, 268]. Recruitment of CCR2^+^ monocyte-derived macrophages appears to promote fibrosis, e.g., via the release of profibrogenic mediators such as TGF-β [264, 265]. Moreover, the subpopulation of TREM2-expressing lipid-associated macrophages is important for the efferocytosis of dying hepatocytes and repair [267, 268]. Failure of TREM2-dependent efferocytosis and repair exacerbates fibrosis, most likely via increased HSC activation [268]. The upregulation of “don’t-eat-me” signals” via CD47 on hepatocytes and its receptor anti-SIRPa on macrophages can lead to a similar suppression of efferocytosis and increased fibrosis in MASLD [80]. In addition to signals from immune cells to HSCs, HSCs also provide signals to immune cells that may contribute to MASLD pathogenesis and possibly regression [236]. HSCs secrete mediators, sometimes termed “stellakines”, in MASLD, including CCL2, CCL11, CXCL10, CXCL12, and CXCL16, which may also increase their recruitment and interaction with immune cells and thereby promote liver fibrosis or repair [223, 246]. Additional proinflammatory signals in MASLD are mediated by microbe-associated metabolic patterns (MAMPs). Because of its direct connection to the intestine via the portal vein, the liver is the first target of gut-derived nutrients and mediators. In MASLD, the gut‒liver axis becomes severely disturbed, and increased exposure to intestinal MAMPs can activate TLRs on hepatic immune cells and HSCs to promote inflammation, immune cell recruitment, and fibrogenesis [269, 270]. Restorative macrophages play a key role in degrading the extracellular matrix and restoring normal liver architecture via their high expression of matrix metalloproteinases [271–274]. It remains to be investigated how HSCs or other liver cell types contribute to the recruitment of monocytes and their polarization toward a restorative phenotype during fibrosis regression. In summary, macrophage‒HSC interactions are central for the pathogenesis and resolution of MASLD, with additional roles for HSC interactions with other immune cells, such as lymphocytes and NK cells, as reviewed elsewhere [237, 246].

### Hepatic stellate cells and their interactions with immune cells in liver cancer

Hepatocellular carcinoma (HCC) represents one of the deadliest complications of chronic liver disease and causes more than 800,000 deaths per year worldwide [275–277]. Almost 90% of hepatocellular carcinomas (HCCs) develop in fibrotic or cirrhotic livers [277]. Accordingly, fibrosis represents a significant risk factor for the development of HCC in patients [261]. HSCs contribute to a tumor-promoting environment in the chronically injured liver through multiple mediators. HSC-secreted type I collagen promotes a stiff environment that activates mechanosensitive signals in the liver, such as TAZ in hepatocytes, and thereby contributes to hepatocarcinogenesis [278]. The key role of type I collagen in HSCs and TAZ in hepatocytes in hepatocarcinogenesis has been demonstrated via cell-specific knockout studies [224]. A number of additional HSC mediators regulate the development of HCC. For example, the production of hyaluronic acid also promotes the development of HCC but in a less potent manner than type I collagen does [224]. Conversely, HSC-secreted HGF protects against the development of HCC by reducing hepatocyte death and the ensuing vicious cycle of regeneration and inflammation [224]. In addition to promoting HCC, HSCs also promote the growth of cholangiocarcinoma and desmoplastic liver metastases [279, 280]. However, this process is not mediated by type I collagen but rather through the secretion of growth factors such as HGF and hyaluronic acid [278]. In addition to affecting the ECM and growth factors, HSCs influence liver carcinogenesis by controlling inflammation and immunity in the liver [278]. In chronic liver disease, there may be an imbalance between increased inflammation and suppressed immunity [224]. HSCs suppress antitumor immunity in liver cancer through a wide range of mechanisms and mediators. The expression of TGFβ and PD-L1 by HSCs and HSC-derived cancer-associated fibroblasts (CAFs) may downregulate T-cell-mediated immune responses and increase regulatory T cells [236, 251, 252, 281–283]. Moreover, HSCs may participate in the mobilization of myeloid-derived suppressor cells via cyclooxygenase-2 [284].

## Endothelial cells and their interactions with immune cells in health and disease

Endothelial cells play a crucial role in homeostasis in the liver [285, 286]. Indeed, angiocrine Wnt signaling establishes and maintains the pericentral phenotype of hepatocytes, as demonstrated in a mouse model in which the Wnt cargo receptor Evi (Wls), which is important for Wnt ligand exocytosis, was deleted in Stab 2^+^ endothelial cells [287]. In line with this study, pericentral angiocrine R-spondin 3 (Rspo3) maintains the pericentral metabolic signature of most pericentral WNT^high^ hepatocytes through β-catenin [288]. However, this conclusion was reached after Rspo3, which is not an endothelial cell-specific Cre driver, was deleted in CAGCreERT2 mice. A recent study using HSC-specific Rspo3 and EC-specific approaches revealed distinct roles for HSC- and EC-derived Rspo3 in zonation and assigned most hepatocyte-regulatory functions to HSC-derived Rspo3 [19]. In the adult liver, endothelial cells appear to be zonated. scRNA-seq revealed that periportal LSECs express high levels of CD36 and Adam23, which progressively diminish in the midzonal and pericentral zones. On the other hand, pericentral LSECs and central venous ECs abundantly express Wnt2, Wnt9, Lhx6, and Kit, which decrease toward midzonal and periportal zone ECs. Flt4, Lyve1, Cd32b, and Stab 2 are highly expressed by LSECs but not by vascular ECs [18, 289–293]. In addition to LSECs and venous ECs, lymphatic endothelial cells (LyECs) also play important roles in the liver, as they maintain fluid balance and immune regulation [294]. Like LSECs, liver LyECs are characterized by the appearance of fenestrae and cellular pores [295]. However, a recent study demonstrated that LyECs in the liver can be differentiated from other ECs through the expression of LyEC-specific IL7 [295], opening more possibilities for studying LyECs in health and disease.

### Role of liver sinusoidal endothelial cells in hepatic immune regulation during homeostasis

**Fig. 4:**
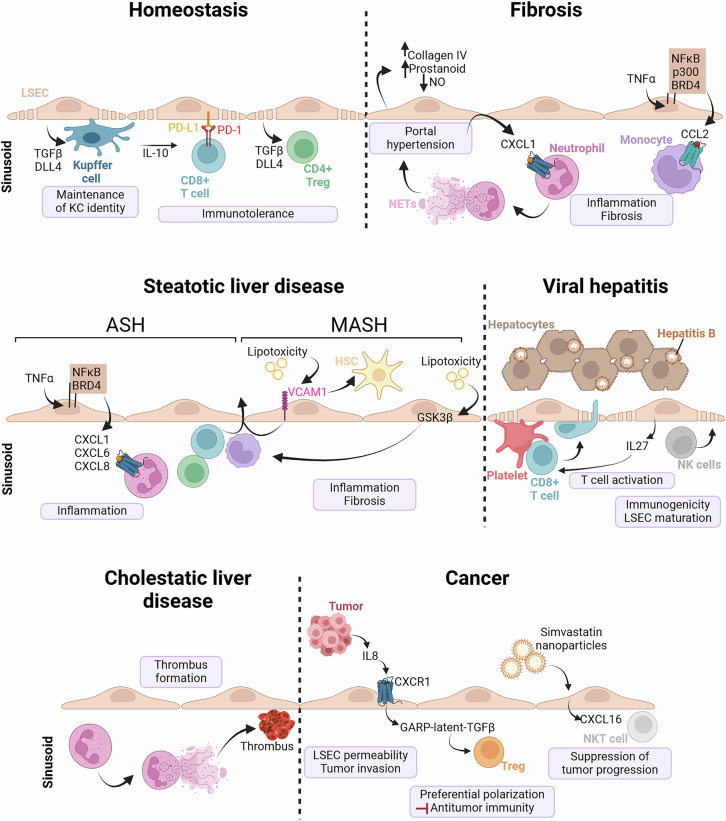
Role of the interaction between LSECs and immune cells during liver health and disease. During homeostasis, fenestrated LSECs maintain Kupffer cell identity through TGFβ and DLL4. LSECs participate in immunotolerance through PD-1/PD-L1-mediated interactions with CD8^+^ T cells and TGFβ and DLL4 release toward CD4^+^ T cells. During liver fibrosis, LSECs lose their fenestrae and produce high levels of collagen IV and prostanoids and low levels of nitric oxide (NO) to increase portal hypertension. In addition, LSECs recruit immune cells that amplify fibrosis through multiple mechanisms. LSECs release CXCL1 to recruit neutrophils, which release NETs and subsequently amplify portal hypertension. In response to TNFα, LSECs also produce CCL2 through an NFκB/p300/BRD4-dependent mechanism to recruit monocytes/macrophages and increase liver fibrosis. During ASH, they release CXCL1, CXCL6, and CXCL8 through an NFκB/BRD4-dependent mechanism to recruit immune cells. During MASH, LSECs respond to lipotoxicity by increasing their expression of VCAM1 to recruit immune cells and activate HSCs. Lipotoxicity also increases fibrosis and inflammation through GSK3β. During viral hepatitis, LSECs interact with platelets to recruit CD8^+^ T cells, which send their protrusions through LSEC fenestrae to reach antigen-presenting hepatocytes. The release of LSEC-derived IL-27 promotes T-cell activation. Intrahepatic T-cell responses can also be enhanced by the natural killer (NK) cell-dependent maturation of LSECs as a beneficial process to control HBV replication and expression. During cholestatic liver disease, neutrophil infiltration in the liver promotes the formation of NETs, which induce the production of procoagulants in LSECs, which are important for the formation of thrombi. Finally, during cancer, tumor cells produce IL-8, which interacts with LSEC CXCR1, leading to the release of GARP-latent-TGFβ, which preferentially induces the polarization of Tregs and inhibits antitumor immunity. Similarly, the uptake of SV-enriched nanoparticles by LSECs leads to the release of CXCL16, recruitment of NKT cells, and suppression of tumor progression. ASH alcohol-associated steatohepatitis, KC Kupffer cells, LSEC liver sinusoidal endothelial cells, MASH metabolic dysfunction-associated steatohepatitis, NET neutrophil extracellular trap, NK cells natural killer cells, NKT cells natural killer T cells, NO nitric oxide

LSECs are the port of entry to the liver for circulating immune cells and can function as resident antigen-presenting cells, promoting immunotolerance under physiological conditions [296]. During homeostasis, LSECs secrete TGF-β and delta-like ligand 4 (DLL4) to maintain Kupffer cell identity (Fig. 4) [297, 298]. In a transgenic model in which Kupffer cells are depleted via diphtheria toxin, LSECs and HSCs recruit circulating monocytes, which acquire Kupffer cell identity [299]. These studies demonstrate the importance of LSECs in maintaining the pool of Kupffer cells in the liver. In turn, Kupffer cells release IL-10 to induce T-cell immunotolerance [300]. CD8^+^ T-cell tolerance is also promoted by LSECs. Indeed, the interaction between antigen-presenting LSECs and naïve CD8^+^ T cells increases B7-homolog 1/programmed death-ligand 1 (B7-H1/PD-L1) expression on LSECs and PD-1 expression on T cells in vitro [301]. This leads to CD8^+^ T-cell tolerance, which is stable when LSEC-tolerant T cells are transferred to congenic animals in vivo [301]. The systemic effect of hepatic tolerance on controlling inflammatory or autoimmune diseases can be explained by the capacity of the liver to generate antigen-specific CD4^+^ CD25^+^ FOXP3^+^ regulatory T cells (Tregs). LSECs are the most efficient cells in the liver that induce Treg generation through TGFβ [302]. In addition to their role in immune tolerance, LSECs play important roles in immune surveillance. In the case of viral infection, major histocompatibility complex class I (MHC-I) molecules can be transferred from HSCs to LSECs, which cross-present soluble antigens to CD8^+^ T cells [303]. These studies have opened an avenue for investigating the detailed mechanisms by which LSECs regulate immunotolerance and immunosurveillance, as well as the balance between these two states.

### Endothelial cell and immune cell crosstalk in the injured and fibrotic liver

The zonation of LSECs, as well as their communication with other cell types, is altered during liver injury. In healthy livers, LSECs prevent HSC activation [304]. In cirrhotic mouse livers, LSECs become capillarized mostly in the pericentral area, where they decrease some of their markers, such as lymphatic vessel endothelial receptor 1 (Lyve1), cluster of differentiation 32b (Cd32b), and Fms-related tyrosine kinase 4 (Flt4) [293]. Capillarized LSECs (Fig. 4), characterized by high nuclear factor kappa-light-chain-enhancer of activated B cells (NF-κB)-dependent collagen IV release [305], lose their HSC-suppressive properties and thereby contribute to an environment that favors HSC activation and fibrosis [304]. Moreover, they secrete vasoconstrictors such as prostanoids and reduce NO release, leading to increased portal pressure [306, 307]. This elevation of blood pressure in the portal vein, named portal hypertension, is a complication of chronic liver disease and is often characterized by high pressures in the liver sinusoids. This mechanical force exerted on LSECs has been demonstrated to increase the release of C-X-C chemokine ligand 1 (CXCL1), as shown in a mouse model of inferior vena cava ligation [308]. CXCL1 secretion, which is mediated by stiffness-dependent glycolysis [309], leads to neutrophil recruitment to the liver. Neutrophils form extracellular traps and microthrombi that, in turn, amplify portal hypertension [308]. During fibrosis, LSECs also recruit monocytes/macrophages to the liver through the release of C‒C motif chemokine ligand 2 (CCL2) [231, 310]. Indeed, in CCl_4_-mediated chronic liver injury, TNFα in LSECs leads to the interaction of NF-κB with histone acetyltransferase protein 300 (p300) and bromodomain containing 4 (BRD4) to epigenetically upregulate CXCL1 and CCL2, which significantly increases liver inflammation and promotes fibrosis. These studies demonstrate that LSECs are crucial for the recruitment of immune cells during liver fibrosis.

### Endothelial cell and immune cell crosstalk during steatotic liver disease

Liver inflammation, along with excessive lipid accumulation and liver fibrosis, are the main features of alcohol-associated liver disease (ALD) and metabolic dysfunction-associated steatohepatitis (MASH). Alcoholic hepatitis (AH) is the most severe manifestation of ALD and presents an accumulation of neutrophils in the liver [292, 311]. Indeed, tumor necrosis factor alpha (TNFα) augments the expression of several chemokines in LSECs, including CXCL1, CXCL6, and CXCL8, through NF-κB and bromodomain-containing protein 4 (BRD4) binding to a common superenhancer (Fig. 4) [312]. Pharmacological inhibition of BRD4 in a murine model of AH reduces chemokine expression and neutrophil infiltration in the liver [312]. In addition to neutrophils, other innate and adaptive immune cells infiltrate the liver during AH [313]. However, the LSEC-dependent mechanisms that attract these immune cells remain to be studied. The proinflammatory phenotype of LSECs has also been explored in MASLD and MASH, where they are characterized by a loss of fenestrae and increased expression of adhesion molecules [292]. Compared with steatohepatitis, the loss of fenestrae has been observed to occur to a greater extent in human steatotic livers [314], suggesting that LSEC capillarization might occur preferentially during the early stages of MASLD. During MASH, lipotoxic stimulation leads to increased expression of endothelial vascular cell adhesion molecule 1 (VCAM1) [315]. The inhibition of VCAM1 in a murine model of MASH leads to decreased HSC activation and fibrosis [316]. Selective deletion of VCAM1 in LSECs in a murine model of CCl_4_-induced liver fibrosis also reduces macrophage accumulation in the liver [316], suggesting that LSEC VCAM1 is important for immune cell infiltration and liver fibrosis. In line with these studies, LSEC injury induced by lipotoxic palmitate leads to increased phosphorylation of glycogen synthase kinase 3 beta (GSK3β). Pharmacological GSK3β inhibition reduces neutrophil and monocyte migration in vitro and ameliorates liver inflammation and fibrosis in a murine model of MASH in vivo [317]. In addition to innate immunity, a recent study demonstrated that CD8^+^ T-cell clonal expansion is a feature of MASH [318], indicating a possible role for CD8^+^ T cells during MASH progression. Nevertheless, a deeper understanding of the molecular mechanism by which LSECs recruit adaptive and innate immune cells during steatotic liver disease is needed. Finally, the accumulation of lipid droplets in the liver generates mechanical forces, and their effect on the biology of LSECs remains to be explored.

### Endothelial cell and immune cell crosstalk during viral hepatitis

Crosstalk between LSECs and CD8^+^ T cells has also been studied in the context of viral hepatitis (Fig. 4). In a transgenic mouse model of hepatitis B virus infection, in which mouse GP-Ibα is replaced with transgenic human GP-Ibα, it has been observed that effector CD8^+^ T cells arrest within the sinusoids by docking on platelets, which are already adhered to LSECs. Then, effector CD8^+^ T cells crawl along the sinusoids and extend their cytoplasmic protrusions through LSEC fenestrae to search for antigens on hepatocytes [319], suggesting a crucial role for LSECs in the process of viral antigen detection by T cells. In addition, HBV exposure leads to a switch from a tolerogenic state to an immunogenic state in LSECs. This process involves the release of LSEC-derived cytokines, such as IL-27, which promote the activation of T cells [320]. Intrahepatic T-cell responses can also be enhanced by the natural killer (NK) cell-dependent maturation of LSECs as a beneficial process to control HBV replication and expression [321]. The modulation and leveraging of the immunogenic versus tolerogenic properties of LSECs, especially in the recruitment and activation of T cells, to treat viral hepatitis seem to be attractive strategies and deserve increased attention.

### Endothelial cell and immune cell crosstalk during cholestatic liver disease

Tolerogenic LSECs have been explored in a model of autoimmune cholangitis. In a model of CD8^+^ T-cell-mediated autoimmunity, the administration of nanoparticles loaded with an autoantigen peptide leads to cross-presentation of the autoantigen on MHC I molecules on LSECs, subsequently reducing T-cell infiltration into the liver and ameliorating cholangitis [322]. Cholestatic liver disease is accompanied by infiltration of neutrophils into the liver, which promotes the formation of neutrophil extracellular traps (NETs) (Fig. 4) [323]. In turn, NETs induce the production of procoagulants in LSECs, which are important for the formation of thrombi [323]. The crosstalk between LSECs and immune cells in cholestatic liver disease remains largely unknown and warrants further investigation.

### Endothelial cell and immune cell crosstalk during drug-induced liver injury

In addition to cholangiopathies, LSECs are also important during drug-induced liver injury (DILI), where they can be a direct target of acetaminophen toxicity, preceding hepatocyte injury [324]. More recently, the G protein-coupled receptor cysteinyl leukotriene receptor 1 (CYSLTR1) was shown to promote acetaminophen-induced injury. CYSLTR1 is expressed in LSECs and monocytes, and its inhibition in conjunction with a G protein-coupled bile acid receptor 1 (GPBAR1) agonist potently reverses LSEC/monocyte interactions and reduces liver damage [325]. It would be interesting to understand how G protein-coupled receptors affect the crosstalk not only between LSECs and monocytes but also with other immune cells.

### Endothelial cell and immune cell crosstalk during liver cancer

The LSEC phenotype, expression profile, and ability to interact with other cells are affected during liver cancer (Fig. 4). Indeed, in mice, the initial steps triggering HCC correlate with LSEC capillarization [326]. During hepatocellular carcinoma (HCC) progression in humans, LSECs lose the expression of their markers, including lymphatic vessel endothelial hyaluronan receptor-1 (LYVE-1), CD32b, stabilin-1, and stabilin-2 [327]. Additionally, LSECs participate in vascular invasion and local microenvironmental immune escape in HCC [328]. In this context, HBV-associated tumor cells release high amounts of interleukin 8 (IL-8), which binds to endothelial CXCR1, leading to increased LSEC permeability to facilitate tumor invasion. The IL-8/CXCR1 axis also induces glycoprotein-A repetitions predominant (GARP)-latent-TGFβ in liver sinusoidal endothelial cells to subsequently provoke preferential regulatory T-cell polarization to suppress antitumor immunity [328]. In line with these studies, a subset of tumor-associated endothelial cells secretes CXCL12, which inhibits the differentiation of CD8^+^ naïve T cells into CD8^+^ cytotoxic T cells and thus mediates immunosuppression [329]. In addition, LSECs alter the DC-mediated activation of T cells, inhibit CD8^+^ T-cell cytotoxicity, and promote T-cell exhaustion in a mouse model of early HCC development [330]. However, LSECs can also be modulated to increase antitumor immunity. Indeed, the upregulation of CXCL16 in LSECs through the administration of simvastatin-loaded nanoparticles in mice leads to reduced LSEC capillarization and the recruitment of NKT cells to suppress tumor progression [331], suggesting that LSECs are promising therapeutic targets. Studies to better understand the modulation of LSEC behavior and tolerogenic versus immunogenic properties, as well as how different LSEC subpopulations interact with immune cells and other cell types in health and liver diseases, are fundamental for the development of efficient therapeutic strategies.

## Looking for a needle in the haystack—the power of single-cell-resolved analyses and data integration for the study of liver inflammation and immunity

Fast-paced recent advances in single-cell-resolved technologies have allowed the scientific community to dissect the liver cellular landscape with unprecedented depth, particularly highlighting the previously overlooked intricate cellular crosstalk between the liver and immune cells [332–334]. More importantly, those technologies have allowed us to “look for a needle in a haystack”, hepatocytes representing the haystack in this analogy, and needles rare populations such as BECs, endothelial cells, HSCs, and rare immune cell populations. Indeed, previous investigations using, for example, RNA bulk sequencing or even tissue microdissections often led to an overwhelming contribution of hepatocyte-derived signals in transcriptomic or proteomic analyses and often completely lacked information on rare cell populations (which often also contain much less RNA and protein than hepatocytes do). One landmark study described the fibrotic niche in healthy and cirrhotic human livers via single-cell RNA sequencing [335]. In addition to providing a first-of-its-kind liver cell atlas, this study revealed the presence of two disease-associated endothelial cell populations in the fibrotic niche, characterized as CD34^+^PLVAP^+^VWA1^+^ and CD34^+^PLVAP^+^ACKR1^+ populations^. Moreover, this same study identified PDGFRA as a marker of scar-associated fibroblasts in cirrhotic livers. The authors’ analysis also provides in-depth characterization of liver macrophages and intrahepatic cellular interactomes and remains highly valuable and illustrates the many opportunities that arose for single-cell-resolved studies [335]. Another multidimensional analysis demonstrated that in a murine model of persistent hepatitis B virus infection, LSECs increased protein kinase A activation on CD8^+^ T cells, which abrogated their ability to perform cytotoxic functions toward infected hepatocytes and, ultimately, failure of virus-specific immunity. This mechanism was termed “the liver immune rheostat” by the authors and further illustrates the complex cellular interplay taking place in liver diseases [336]. Similarly, a combination of single-cell transcriptomics and multiplex immunofluorescence techniques revealed that HSC depletion led to altered hepatocyte zonation and liver repair mechanisms in mice and that HSC R-spondin3 expression was greater in patients with a better prognosis in ALD and MASLD [19]. In line with high-throughput technologies, several recent studies have utilized spatial and single-cell multiomics to investigate the immune landscape of liver cancers, including hepatocellular carcinoma and cholangiocarcinoma [337–339]. These studies elucidated the tumor microenvironment, immune cell populations, and effects of immunotherapies on immune correlates in patients with liver cancer. Integration of multiomics analysis can provide a more complete landscape of the immune mechanisms of disease progression. However, recognizing the limitations of single-cell-based and spatial transcriptomic technologies is important for proper data interpretation. For example, single-cell RNA-seq relies on liver digestion and cell isolation and can lead to artifacts caused by prolonged warm digestion, complications for droplet-based RNA sequencing for large cell types (e.g., steatotic hepatocytes) and biases owing to the inability to achieve proper representation of cell types in highly fibrotic livers. As such, single-nucleus RNA sequencing provides less bias and better representation of cell populations such as hepatic stellate cells and hepatocytes from steatotic livers. Likewise, sequencing-based spatial transcriptomic studies often do not achieve single-cell resolution. In addition, bioinformatic tools are rapidly evolving; thus, the resolutions and codes used for data analysis can become obsolete and need constant updating.

In addition, technologies for multiplexed tissue imaging have become broadly accessible and, consequently, have been used. This includes high-complexity platforms such as imaging mass cytometry and sequential multiplex immunofluorescence. This has led to the identification of major disease progression-associated histological changes involving multiple cell types, such as MASLD/MASH, PSC, PBC liver cancer and neonatal infections [175, 340–342]. Multiplex immunofluorescence also holds key advantages for molecular pathway exploration in animal models, as this allows for a multidimensional evaluation of cellular phenomena on archival, formalin-fixed and paraffin-embedded tissue sections [343]. As such, multiplex immunofluorescence revealed the crucial role of Jagged-1-expressing monocyte-derived macrophages in inducing *Sox9* expression in hepatocytes, allowing liver repair mechanisms to engage in a murine model of acute concanavalin-A-induced liver injury [344]. The same study also demonstrated the mobilization of activated HSCs to form a scar around the necrotic area, representing another example of how multiplexed technologies now support the exploration of distinct cellular responses occurring concomitantly. In addition to expanding the investigational toolbox, multiplex immunofluorescence dramatically increases immunostaining-based data robustness, as it allows the experimenters to identify potentially questionable antibody specificities by better visualizing positive signal distributions. This increasing dimensionality in digital images further stimulates the development of advanced tools for image analyses and data extraction that go beyond what the experimenter’s eye, which is perhaps unconsciously biased but surely biologically limited, may see [345]. Intense efforts are being made to deploy artificial intelligence-based algorithms that can facilitate large dataset analyses and data integration, with implications far beyond image analyses or data extraction [346].

Notably, some of the studies mentioned above, despite being of high quality, remain descriptive of several aspects—understandably, since not all mechanisms may be tested by a single group within the time frame of one manuscript and without leading to an overwhelmingly complex report. Nevertheless, groundbreaking advances in the field of in vitro modeling of liver diseases are also taking place, and this is to some extent supported by recent large datasets [347]. On the one hand, the community now benefits from extensive information that can be used to define transcriptomic and proteomic signatures associated with specific cell types and states, which provide essential input as to what models should be mimicked [161]. On the other hand, interactome analyses emphasized the need for multicellular culture systems. Indeed, crucial milestones have been reached within the last decade, from the isolation and in vitro expansion of bile duct-derived progenitor cells to diseased patient-derived cholangiocyte organoid cultures for detailed disease-driving mechanism investigations [161, 348]. These approaches hold promise in applications such as autologous transplant. For example, gallbladder-derived cholangiocyte organoids have been shown to engraft in the intrahepatic biliary tree after injection into deceased transplant donor livers and prevent ischemic injury while the organ is under machine perfusion, which represents a promising example of ex vivo cell-based therapy [164]. This approach using unaltered, autologous gallbladder cells combined with genetic alterations in MHC protein expression may further prevent the recurrence of autoimmune cholangitis after liver transplantation [349]. Further developments are expected in the near future, notably allowing for the generation of patient-derived multicellular and immune-competent organoid systems that will open doors for mechanistic studies of cellular crosstalk [350]. Moreover, we and others recently developed in vitro, mouse primary cell-based models for the study of immune-driving mechanisms of acute liver injuries and, potentially, drug candidate testing in a multicellular environment [351]. It is expected that such models containing primary human immune and liver cells will be developed in the near future, which could represent major advances in the field of personalized medicine.

However, this must not be underestimated, and conventional and long-term validated methods remain necessary to confirm each single brick of crucial data generated from large-scale methods. As such, in vivo experimentation, monocultures, single immunostaining with robust controls, targeted protein expression quantitation, and targeted molecular pathway interventions, to name a few, are still crucially needed despite the current enthusiasm and, obviously, the many opportunities derived from novel technologies.

## Conclusions

There is increasing evidence that cell‒cell communication involving multicellular circuitries is essential for maintaining proper liver function during homeostasis. Not a single cell in the liver exists and functions properly in isolation, and disease processes in the liver or any other organ involve multiple cell types [352]. Liver disease is often associated with and potentially aggravated by dysfunction of these multicellular units that maintain proper function. Moreover, the liver is a primary example of a highly resilient organ that can efficiently repair and regenerate and deal with a wide range of often harmful or immunogenic substances and pathogens that enter through its dual vascular supply while maintaining metabolic functions that are essential for organismal survival. Given that modern diseases often do not cause massive life-threatening but rather chronic low-grade injury and inflammation, further understanding of multicellular hepatic communication circuitries may provide a deeper understanding of liver regeneration and restoration, which represent unmet needs in the field. Interactions of liver cells with immune cells are not only key for many aspects of liver pathogenesis but also particularly important and promising for restorative therapies, as demonstrated by recent trials [271]. Similarly, multicellular circuits involving HSCs, LSECs, hepatocytes and Kupffer cells maintain proper liver function in homeostasis and become deranged in liver disease [19, 225]. Promoting homeostatic and restorative properties in these multicellular circuits may hold great therapeutic promise and synergize with current therapeutic concepts that are largely aimed at curing the causative agent but often fail to restore liver architecture and function in advanced disease.
